# Supplementary material for: DMRforPairs: identifying Differentially Methylated Regions between unique samples using array based methylation profiles
Source: BMC Bioinformatics. 2014 May 15;15:141. doi: 10.1186/1471-2105-15-141 (PMC4046028; doi:10.1186/1471-2105-15-141)
Supplement: Additional file 2 — DMRforPairs output for the comparison of A431-MCF7 and NA17018-NA17105. Please start from the HTML files in each folder. Available via the BMC Bioinformatics website. [file 1471-2105-15-141-S2.zip › 1394847754114233_MOESM2_ESM/A431_MCF7/figures/26955.pdf]

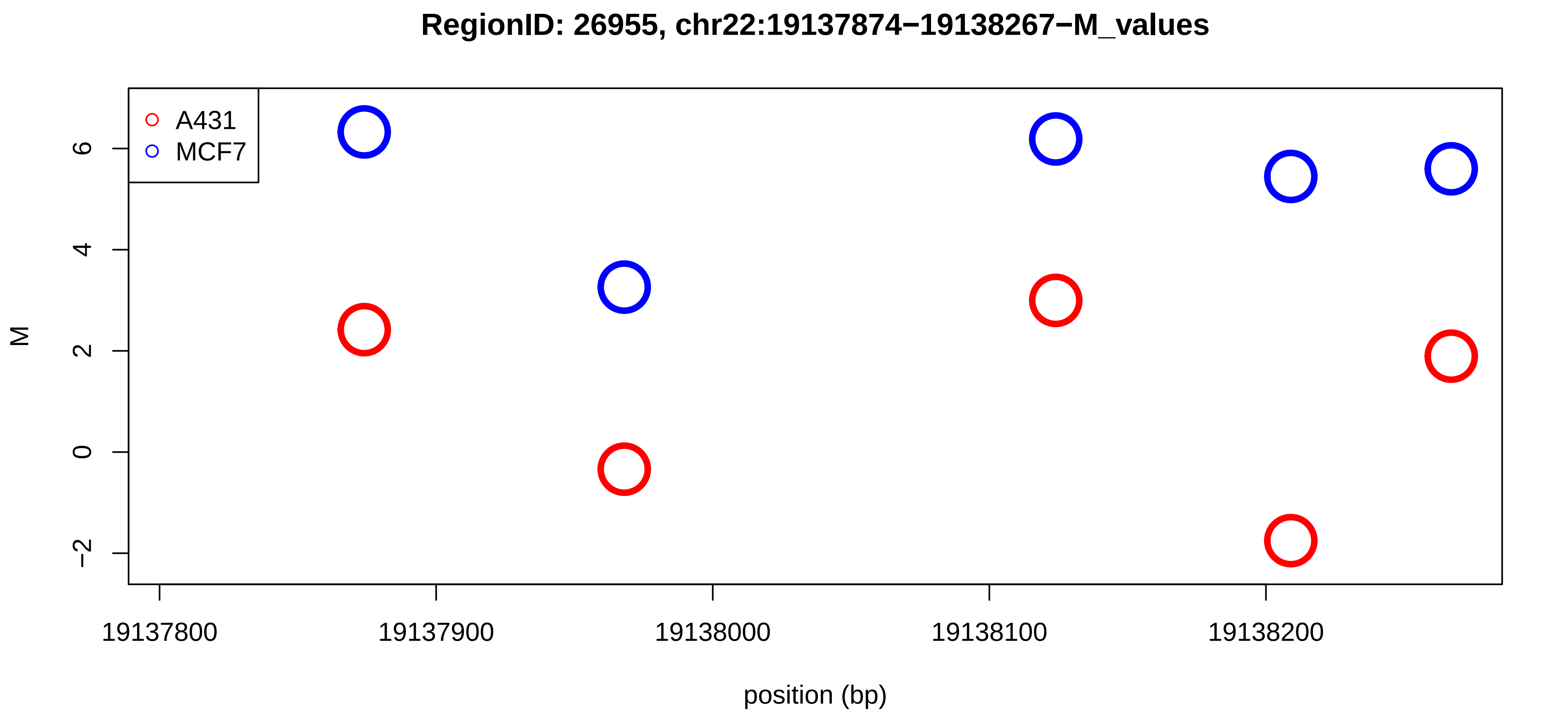
RegionID: 26955, chr22:19137874−19138267−M_values
A431
MCF7
M
6
4
2
0
−2
19137800
19137900
19138000
19138100
19138200
position (bp)

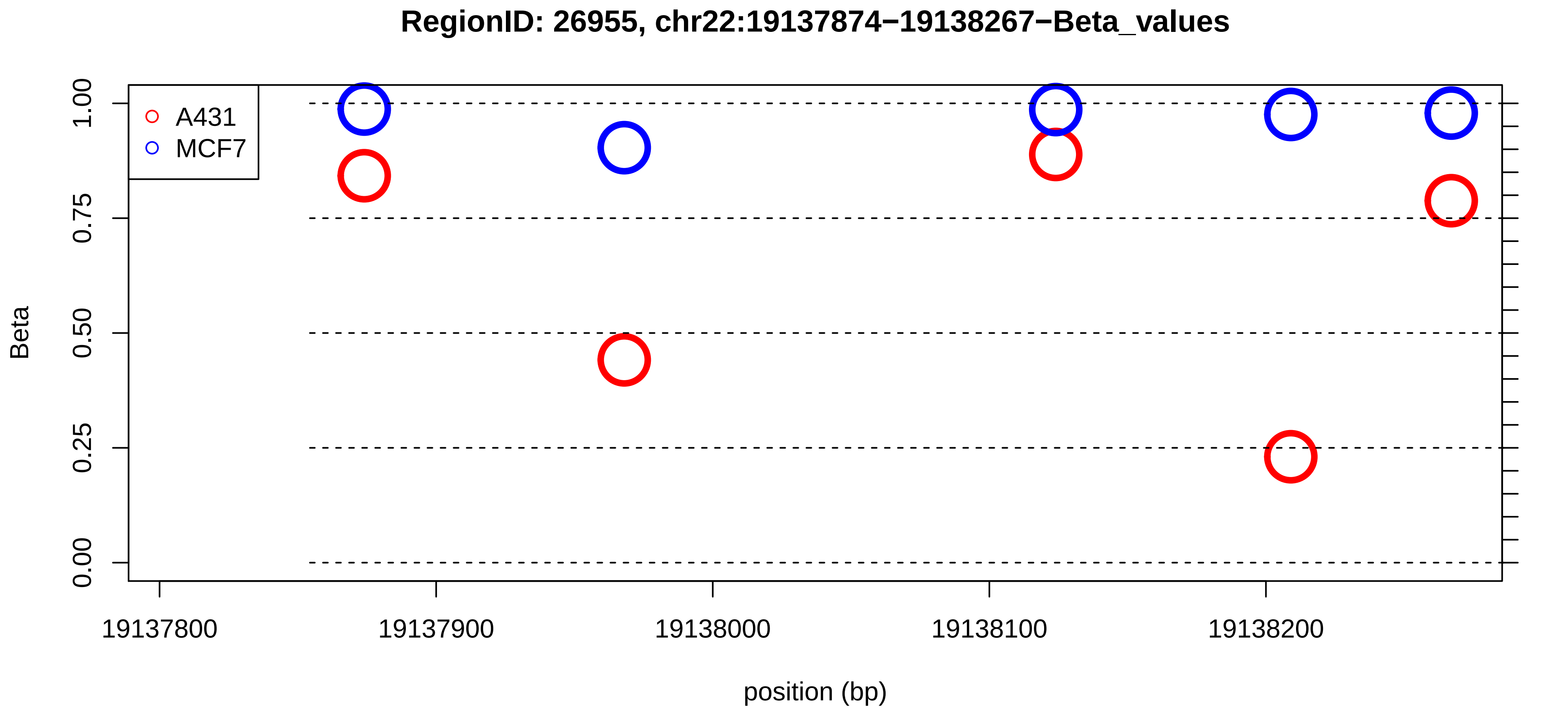
RegionID: 26955, chr22:19137874−19138267−Beta_values
A431
MCF7
Beta
1.00
0.75
0.50
0.25
0.00
19137800
19137900
19138000
19138100
19138200
position (bp)

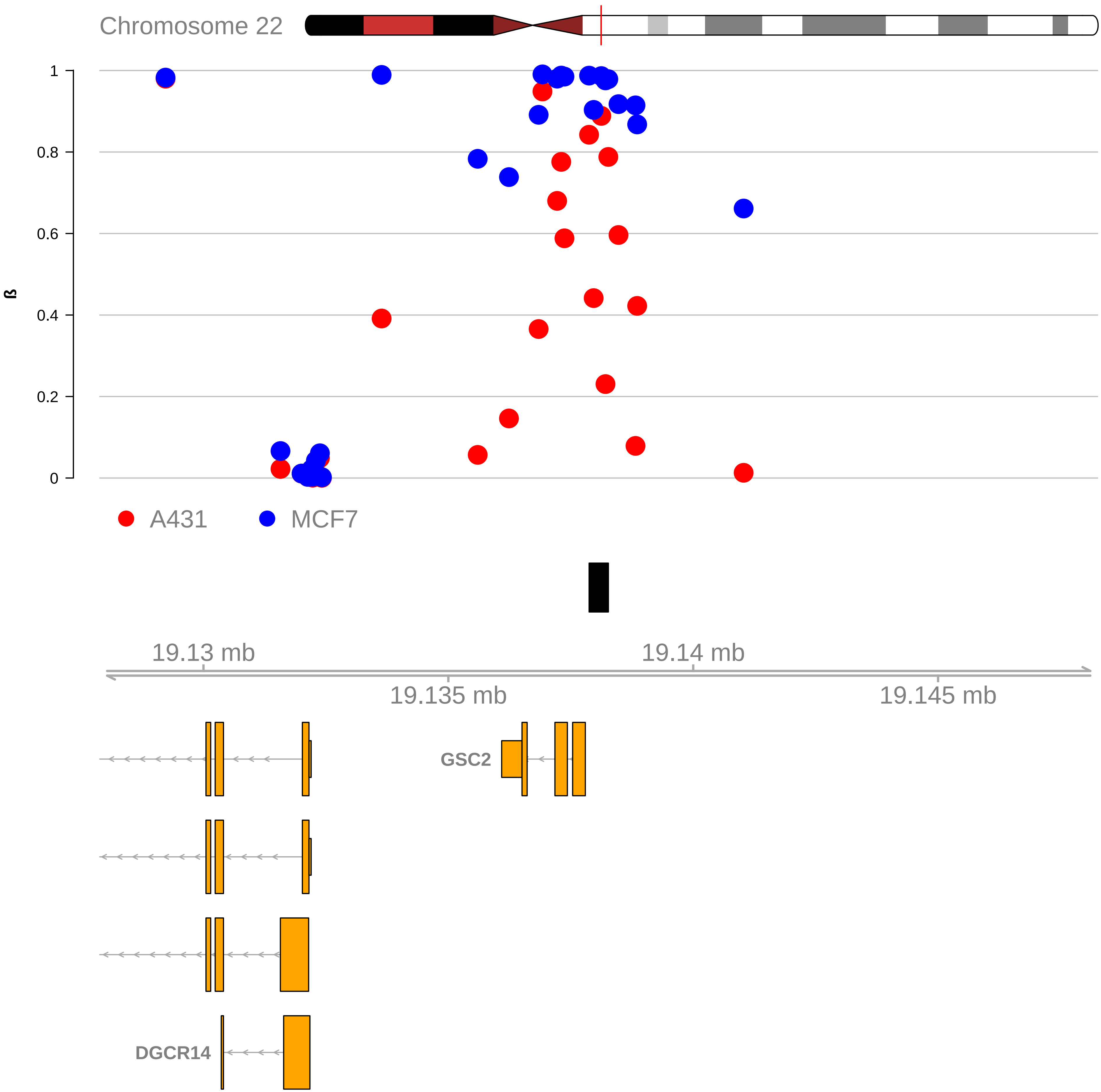

Chromosome 22
ß
1
0.8
0.6
0.4
0.2
0
A431
MCF7
19.13 mb
19.14 mb
19.135 mb
19.145 mb
GSC2
DGCR14
